# Content analysis of patient support groups related to myositis on Facebook

**DOI:** 10.1007/s10067-023-06854-8

**Published:** 2024-01-12

**Authors:** Aiman Perween Afsar, Shounak Ghosh, Renil Sinu Titus, Karen Cheng, Arundati A. Kanawala, Peter Kerkhof, Jessica Day, Latika Gupta

**Affiliations:** 1 Maulana Azad Medical College, New Delhi, India; 2 Department of Rheumatology, Calcutta Medical Research Institute, Kolkata, 700027 India; 3 Seth G.S. Medical College and K.E.M. Hospital, Acharya Donde Marg, Parel, Mumbai, India; 4 Patient with Myositis, Basel, Switzerland and EU Patient Advocacy Lead, Patient-Led Research Team, Myositis Support & Understanding (MSU), Lincoln, DE USA; 5 Smt. Kashibai Navale Medical College, Pune, India; 6 Department of Communication Science, Vrije Universiteit Amsterdam, Amsterdam, Netherlands; 7 Department of Rheumatology, Royal Melbourne Hospital, Parkville, VIC 3050 Australia; 8 Dept. of Rheumatology, Royal Wolverhampton Trust, Wolverhampton, WV10 0QP UK; 9 Division of Musculoskeletal and Dermatological Sciences, Centre for Musculoskeletal Research, School of Biological Sciences, The University of Manchester, Manchester, UK

**Keywords:** Facebook, Muscle, Myositis, Online support group, Rare diseases, Social Media

## Abstract

**Introduction:**

Idiopathic inflammatory myopathies (IIM) are heterogeneous and complex, and routine consultation can be overwhelming for patients, or sometimes so rushed that patients feel unable to discuss their needs and wishes adequately. As a result, online patient support groups (PSGs) on social media platforms like Facebook may help provide them with information they are seeking, and the support of the patient community who are living with this condition. Our goal is to explore the current landscape of PSGs in IIM to discuss the future of such groups and their role in supporting patient-driven self-management of complex connective tissue diseases.

**Methods:**

We investigated factors that influence engagement in publicly accessible support groups on Facebook for patients with myositis. We analysed posts from myositis-related Facebook groups and pages between July 10, 2022, and October 2022. Data were extracted from each post, including presentation format (text, picture, video or mixed media) and content type (news, personal feelings or information). To gauge the post’s impact, we measured engagement metrics, such as likes, comments, shares and reactions.

**Results:**

Nearly three-quarters of the groups were private. Among the open ones, most posts seem to comprise pictures with text. Notably, engagement levels were higher for multimedia posts, with the exception of comments in groups, where engagement was comparatively lower.

In terms of content, the majority of posts fell under the ‘personal’ category, followed by ‘information’ and ‘news’ posts, with information posts in groups receiving the most interactions. Moreover, groups exhibited higher total engagement compared to pages when considering all posts cumulatively.

**Conclusions:**

Our observations indicate that patients with myositis seek information on the condition online, and the multimedia nature of content presentation significantly influences engagement. These digital forums serve as valuable platforms for fostering connections among diverse individuals, providing a perceived safe space for sharing their personal experiences and varied perspectives, and potentially mitigating social isolation.

**Table:** 

**Key Points** • *Patient support groups on myositis are a key source of support and information for patients.* • *Public posts with multimedia content garner the most engagement.* • *The majority of posts are personal in nature, with a smaller proportion of content providing news or information.*

## Introduction

Idiopathic inflammatory myopathies (IIM) are a heterogeneous group of diseases associated with considerable morbidity [1]. Although survival rates continue to improve, the physical and psychosocial challenges faced by patients with myositis can negatively impact their quality of life [2]. Due to the complex, multisystem nature of IIM and the intricacies of immunosuppression(such as the different pathogenesis of different subtypes of IIM, or the multiple therapies used for immunosuppression and their variable responses in different patients), comprehensive medical consultations that address the various facets of the disease are essential to ensure optimal care. Shorter consultation times in overburdened post-pandemic healthcare systems may leave patients and physicians feeling overwhelmed [3] and hinder their understanding of the disease, leading to suboptimal management. Consequently, patients may seek online resources to address their unmet information and support needs ^3^.

Group interventions tend to perform better as a resource for moral support and information [4]. Online patient support groups (PSGs) on Facebook, one of the popular global social media networks, have emerged as valuable sources of moral support and information for individuals with myositis. Many healthcare systems lack the resources which patients need outside of the short consultation time with their primary myositis physician. These online PSGs offer distinct advantages including broad accessibility for patients and survivors at a low cost without spatial, geographic, economic or temporal constraints [2]. They provide the option of anonymity and bring together a variety of individuals with diverse experiences and perspectives. These platforms provide learning opportunities, not only for new and existing members, but also potentially for their social support network and healthcare professionals. PSGs empower patients by providing access to information while facilitating new social networks and decreasing isolation [5, 6]. Therefore, we explored the current landscape of publicly available support groups on Facebook for patients with myositis.

Engagement serves as a direct measure of a post’s relevance to its viewers and contributes to the satisfaction and well-being of both the creator and consumers, fostering a meaningful relationship. As such, it can serve as an excellent indicator of post efficacy [7]. The foundation of patient education is rooted in the ability to generate interest in their unmet needs. In this study, we investigate the factors that influence engagement within publicly accessible support groups on Facebook for patients with myositis.

Our analysis focuses on understanding the factors that impact engagement with a post, considering the role of both content and its presentation, whether in the form of text, pictures or a combination of both. By gaining insights into these aspects, we aim to help organisations and individuals responsible for these platforms to devise strategies that can improve participation within online PSGs when necessary and provide content that resonates more effectively with the patients. Ultimately, our research endeavours to explore the current landscape of PSGs in rare diseases like IIM and to discuss the future potential of such groups and their role in supporting patient-driven self-management of complex connective tissue diseases. We aim to help foster greater patient acceptance and interaction and contribute to improving patient care and support within the myositis community.

## Methods

We conducted a comprehensive search on Facebook using nine predefined search terms, which were adapted from a similar study [8], including ‘Myositis’, ‘Idiopathic Inflammatory Myositis’, ‘Dermatomyositis’, ‘Polymyositis’, ‘Cancer Associated Myositis’, ‘Inclusion Body Myositis’, ‘Immune Mediated Necrotizing Myopathy,’ ‘Juvenile Dermatomyositis’ and ‘Overlap Idiopathic Inflammatory Myositis’. Through this systematic search, we aimed to gather relevant data and insights from Facebook posts associated with these specific myositis-related terms. Our main objective was to determine the posts that patients found most relevant, with engagement as the primary outcome measure.

### Inclusion criteria

Online PSGs were identified by running the above search terms in the Facebook search bar. Groups or pages had to be clearly recognizable as support groups for myositis patients. Groups with English posts were included. Posts from 10th July 2022 to 10th October 2022 were analysed.

### Exclusion criteria

Groups/pages that were not in English, had restricted access or were not related to IIMs were excluded.

### Data extraction

Data extraction was conducted on a single day, October 11, 2022, by three independent reviewers (APA, RST and SG). All reviewers independently reviewed posts from the past 3 months, spanning from July 10, 2022, to October 2022. For each post, we extracted information on its presentation format and content. Differences were resolved by consensus under the supervision of a third investigator (LG).

Content presentation was categorized as text, picture, video or mixed media (e.g. picture with text). The content of each post was analysed and further categorized as news, personal feelings or information. ‘News’ posts included updates on upcoming events, fundraising efforts, new drug or product studies, advertisements and petitions. Posts falling under the ‘personal feelings’ category encompassed personal experiences, expressions of gratitude, words of encouragement, prayers or discussions about social strain. All posts seeking help, asking for information, clarifying doubts or spreading awareness were grouped under the ‘information’ category. Pages were classified based on Facebook-provided descriptions as communities, charity organisations, non-profit organisations, medical and health pages, sportspersons and personal blogs.

### Validation

A secondary search was conducted using a social media content scraper available on https://apify.com/, to characterize the distribution of posts from different users on July 13, 2023. The posts were further classified into the relevant categories as described above.

### Analysis

Descriptive statistics were utilised to analyse the impact of the presentation and the content of the posts by taking into account their engagement, i.e. the sum of likes/reactions, comments and shares. Reactions are the six ways to react to posts with animated emotions love, ha-ha, wow, sad, angry and likes. The sum of engagement was further added up for each group.

## Results

### Groups

**Fig. 1:**
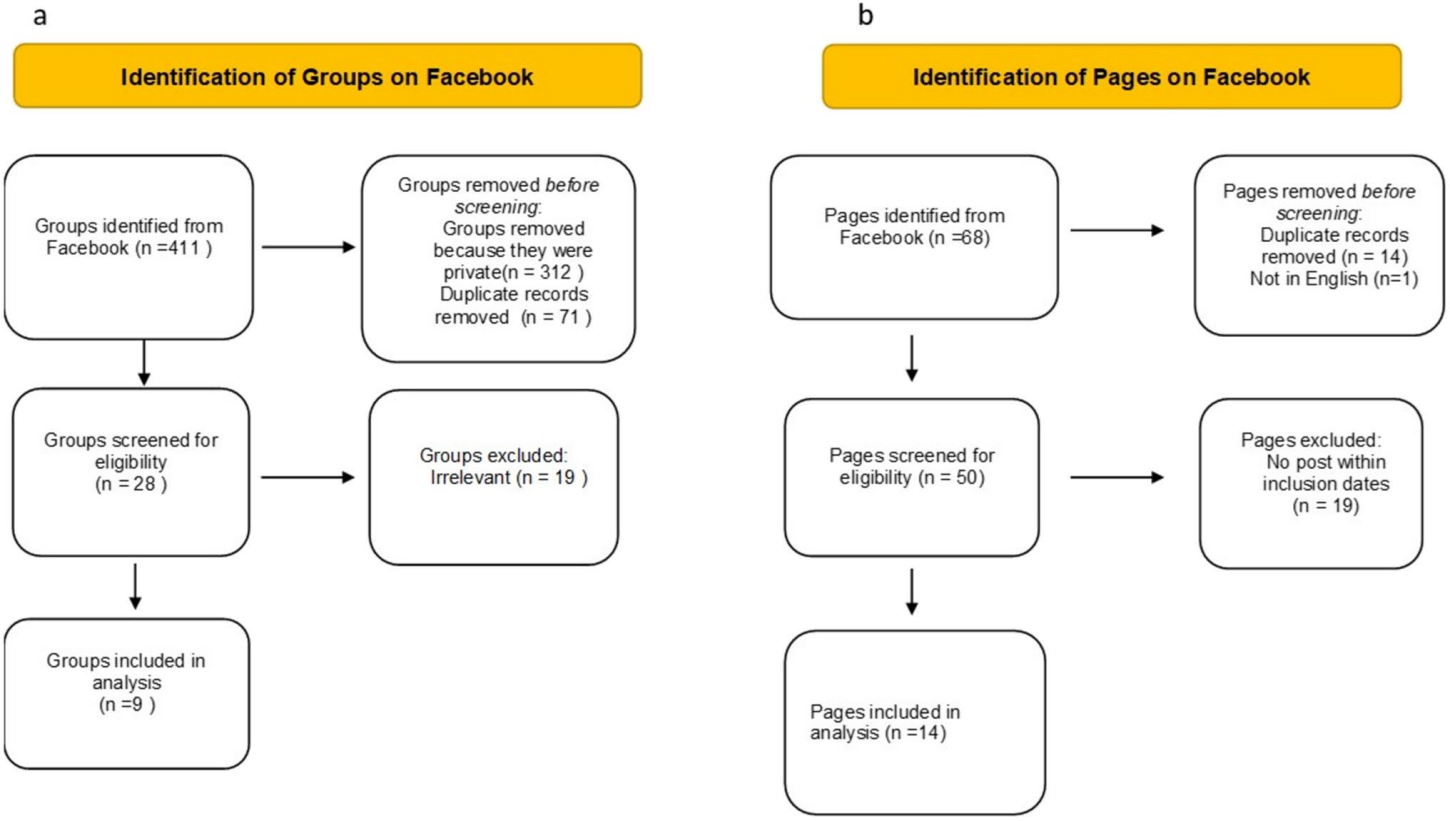
Screening of groups (**a**) and pages (**b**) on Facebook

Our initial searches yielded a total of 411 groups, with 99 being public groups (24%) and the remaining 312 being private (76%). After removing duplicate records (*n* = 71) and unrelated groups (*n* = 19), we selected and analysed nine public, relevant and unique groups for this study (Fig. 1a).

During the study duration of 3 months, from July 10, 2022, to October 10, 2022, the top three groups with the highest number of posts were created in 2008, 2007 and 2019, respectively. On the other hand, the groups with the lowest number of posts were established in 2009, 2017 and 2016 (Table 1).

**Table 1:** Selected publicly available groups and their characteristics

Group name	Location	Total members	No. of admins or moderators	Engagement	Posts in the 3 months of analysis	Creation date
Inclusion body myositis (IBM)	USA	2528	3	14376	539	28-Nov-2008
Inclusion body or myositis (open group)	CANADA	1834	4	1209	197	15-Dec-2007
Myositis warrior	USA	1492	2	7204	350	20-Sep-2019
The myositis foundation	NA	102	1	0	1	12-Nov-2011
Myositis support group	USA	63	1	8	5	10-Feb-2010
Chicago myositis support group	USA	50	1	16	7	3-Nov-2019
Myositis support group — DC, MD, DE and no. VA group	Maryland, USA	45	2	0	1	15-Aug-2016
The Myositis Association, India	INDIA	23	3	1	3	30-Sep-2017
Myositis Support Group of Singapore	SINGAPORE	16	1	2	2	30-Nov-2009

*As of 11 Oct., 2022*

### Pages

Searches yielded a total of six potentially relevant pages. Of these, duplicates (*n* = 14), those not in English (*n* = 1) and those without any post within inclusion dates (*n* = 39) were excluded to yield 14 pages (Fig. 1b). Among them, four were classified as charity organisations, three each as communities and non-profit organisations, two as medical and health pages and one each as a sportsperson’s and a personal blog as displayed on their Facebook pages. The Myositis Association (TMA) had the highest number of followers at 11,000 (Table 2).

**Table 2:** Selected pages and their characteristics

Page name	Category	Followers	Likes	Posts in the study period	Engagement	Creation date
The Myositis Association	Charity Organization	11,000	1814	93	2546	09-May-2009
Cure JM Foundation	Charity Organization	8900	3768	102	4227	03-Nov-2009
Myositis support and understanding—MSU	Charity Organization	7900	275	80	711	04-Feb-2014
Myositis UK	Non-profit organisation	2500	2400	22	628	19-Sep-2013
Cure IBM	Non-profit organisation	1200	31	4	39	08-Nov-2017
Myositis women of colour	Charity Organization	657	635	17	35	28-Aug-2020
Myositis Canada	Non-profit organisation	587	552	3	7	21-Feb-2016
Vee Rob’s Myositis Awareness First Pitch Campaign	Sportsperson	433	418	7	85	08-Apr-2018
Myositis UK—Let’s Meet Up	Community	421	410	1	0	07-May-2014
Steph’s battle with necrotizing autoimmune myositis	Personal Blog	211	NA	3	128	09-Aug-2020
Dan Macri Yoke Record for Myositis	Community	148	143	5	64	08-Feb-2013
TN Myositis Support Group	Medical and health	139	125	22	12	08-Dec-2020
The Myositis Association, India	Health and wellness website	136	133	1	0	30-Sep-2017
Myositis SEA	Community	1	1	2	0	09-Dec-2021

*As of 11 Oct. 2022*

### Type of posts by medium and content

Both groups and pages preferred posts containing both text and photos, resulting in the highest number of posts in this format: 36.8% and 53.1% for groups and pages respectively. Engagement levels, measured by the total number of likes, comments, shares or reactions, were highest for these multimedia posts, with the exception of comments in groups (55% of all comments were for posts with text). Conversely, posts containing only photos or videos received the least engagement and garnered the lowest traction compared to other media types.

**Fig. 2:**
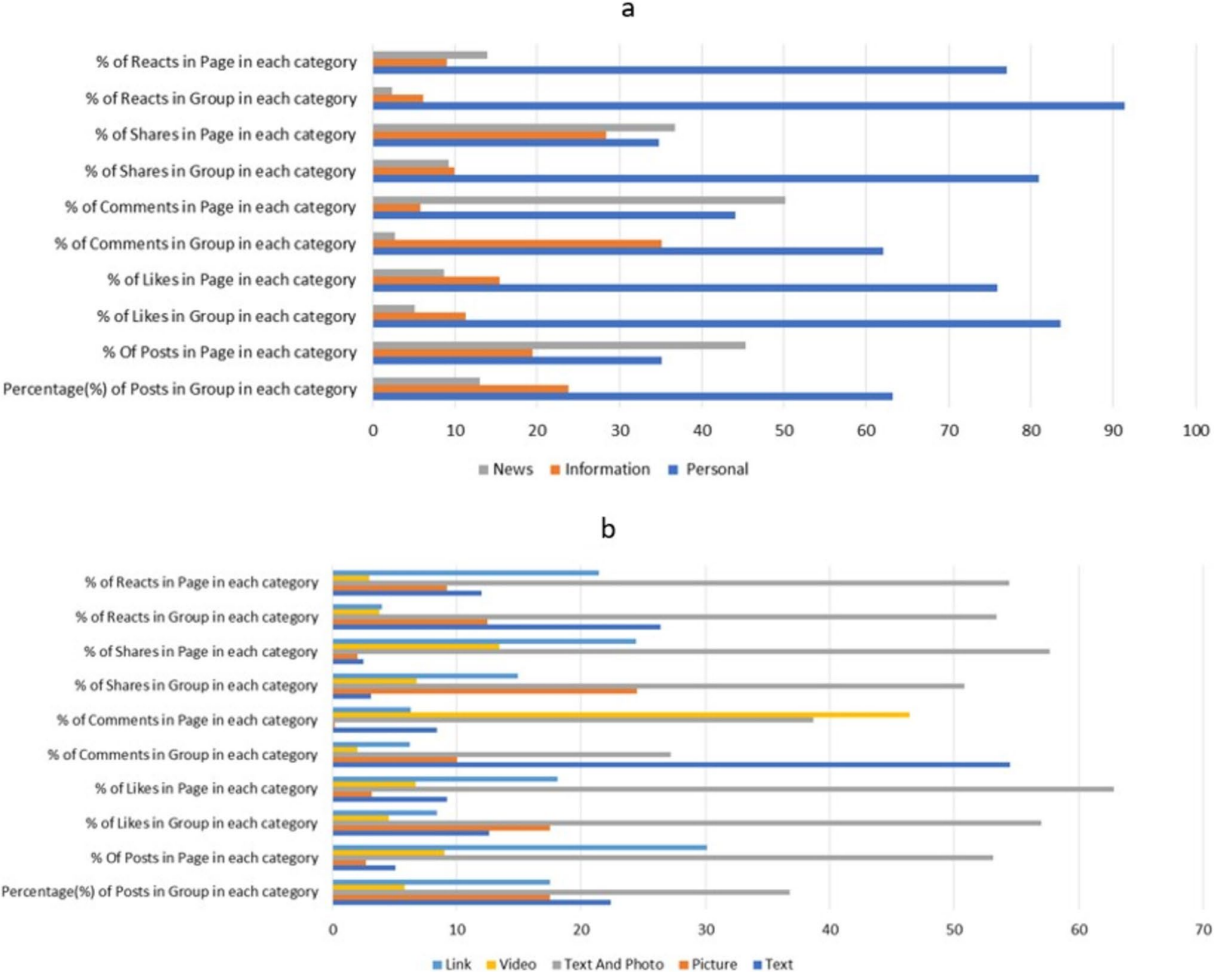
Engagement parameters of posts based on their content (**a**) and visual presentation (**b**)

In terms of content, the majority of posts fell under the ‘personal’ category, followed by information and news posts. On a per-post basis, personal posts within groups or pages received the highest total engagement. However, when analysing comments, information posts in groups received the most interactions (Fig. 2).

### Validation data

Automated searches were conducted using the social media scraper from https://apify.com/ in July 2023 to gather data from the three major groups: Inclusion Body Myositis (IBM), Inclusion Body or Myositis (open group) and Myositis Warrior. Six hundred sixty-four posts were gathered, and almost 70% of the posts were personal in nature. All posts with 50 or more likes had personal content. Interestingly, half the posts (50.7%) related to ‘inclusion body myositis’ were from a single user.

When considering all posts cumulatively, groups consistently exhibited higher total engagement compared to pages. This trend was maintained when stratifying posts by content, except for shares per post for information posts. Overall, groups demonstrated greater engagement across various content types, except in the specific case of information posts’ shares per post.

## Discussion

A large number of groups were available on Facebook. However, the data were publicly available for only a few groups, the majority being private and requiring approved membership for access. This finding aligns with the research of Titgemeyer et al., which emphasises a need for private support spaces for patients with rare diseases [9].

Among the nine publicly available groups explored, engagement and the number of posts in each group were proportional to the number of members. The group with the most members, Inclusion Body Myositis (IBM), had the highest number of posts (539) and maximum engagement (14,376). Conversely, the group with the least number of members, Myositis Support Group of Singapore, had minimal engagement. This indicates that active groups exhibit higher engagement and retention of members (Table 1).

Notably, patients and support providers from developed countries such as the USA and Canada have been more proactive in initiating and maintaining these groups, as evidenced by the location of the top 3 groups by number of posts. The earliest support groups were formed in 2007 and 2008, just 3 years after Facebook was founded. The early emergence of these groups highlights that there was a pressing need for enhanced support for patients with rare diseases, beyond what could be provided by their healthcare practitioner and traditional community services. It also underscores the early recognition of social media platforms as a promising solution by the patient community to reach others for shared support. The data indicates a wide variation in the frequency of posts made in a year. The top 3 active groups had between 197 and 539 posts in the study duration spanning 3 months, indicating a high level of activity. Groups from Asia seemed less active.

Similarly, relevant pages related to myositis appeared in 2009, within 2 years of the availability of ‘pages’ on Facebook. These exhibited similar content profiles and engagement as groups.

It is noteworthy that a significant number of individuals felt comfortable sharing their feelings or experiences within publicly available Facebook myositis support forums, with nearly half of the posts being about an experience or feeling. Notably personal posts also fostered most engagement, suggesting this being of special value for the patient community while seeking support online. Posts in the form of text received the greatest number of comments in groups (two-thirds of the total comments). The posts containing only text comprised personal experience or information-seeking content. The significantly higher number of comments indicates the willingness of fellow members to provide help. Multimedia posts or posts containing a video receive the most reacts or comments on the pages. Overall, content presentation in the form of text along with a picture received maximum engagement. This is similar to the study by Subirats et al. where Facebook analysis showed that posts with pictures and positive comments had the highest engagement [10].

Interestingly, an independent assessment by one of the authors revealed that half of the posts on inclusion body myositis were by a single user, thus underlining the importance of using social media as a personal platform for advocacy. An awareness of the coercive power of social media algorithms is important in the post-truth era [11], where a ‘spiral of silence’ may be created [12] that favours one opinion over another. One must interpret the themes of social media content with a filter to reduce such biases, in order to obtain a true picture of patients’ needs.

Groups exhibited a higher number of likes, comments and reacts per post compared to pages, supporting the notion that individuals participating in groups experience a heightened sense of community and connection with other group members. Online support groups provide a platform for individuals to exchange different kinds of support, and studies by Steadman et al suggest that informational and emotional are the most frequently provided support. Network support also emerges, whereby members use the support group as a common meeting ground and where all issues relating to a specific illness or problem can be openly discussed [5].

Contrary to a study focusing on Facebook groups for multiple sclerosis communities where information and awareness-raising posts comprised around 70% of the total content, we observed that nearly one in five posts in the myositis community was information-related [13]. Recent content analysis of myositis on YouTube iterates the relevance of valid and reliable medical information shared across platforms [8], as inactive members of online forums may benefit from the shared knowledge and experiences without active engagement via reacts or posts/comments [14].

PSGs seem to be valuable resources to provide community support for individuals suffering from IIM. Further research evaluating the quality of content, particularly medical content distributed within online PSGs, and the unmet needs of patients may provide valuable insights. Furthermore, online networks may serve as valuable portals through which a larger and more diverse patient population can be accessed for research purposes, thereby enhancing our understanding of the disease [15].

More recent information on ‘medical gaslighting’ in both the medical profession and on social media platforms heeded a call for members of social media groups to exercise precaution in interactions with other members. There is a call to ensure that the psychological safety of people who are already vulnerable from their physical condition be protected from further psychological harm from others who may not have their best interests and well-being in mind during any kind of communication, online or in person [16].

Our methodology explicitly outlines that our search was limited to publicly accessible groups and pages. The absence of data on removed/deleted posts, coupled with our adherence to specified timeframes, may limit generalisability of these observations. While content in these public groups may be moderated by administrators, there is currently no accessible method for users to verify such moderated content.

However, this is one of the first studies to explore the landscape of online patient support groups focused on myositis, shedding light on their content, geographical distribution, membership numbers and levels of engagement with respect to the number of posts and the reacts, likes, comments and shares they received. We fully acknowledge the limitations of our study, including the exclusion of private and non-English groups and the cross-sectional nature of analysis limiting the ability to identify trends over time. Moreover, engagement observed on social media platforms may not necessarily reflect the perceived benefit of these forums for patients to manage their condition. This study caters to myositis but did not include information on anti-synthetase syndrome; some patients with the condition may sometimes have myositis.

## Conclusion

This pilot exploration offers preliminary insights into the potential of these communities to offer mutual support and understanding. Online PSGs hold significant promise as a means of reaching a broader population of patients, survivors and care providers, providing both mutual education and support. These forums facilitate connections between diverse individuals, provide a space for individuals to offer different perspectives and experiences and may potentially decrease social isolation.

Online PSGs have emerged as a valuable platform to reach a large and diverse audience, while providing anonymity (if desired) and hence creating a perceived safe space for patients to interact and seek support. This is an evolving area, and more research is required to explore best practices, the impact of facilitator or moderator involvement, the quality of posted content and the effectiveness of these groups in providing support.
